# Effect of operational parameters on the performance of an anaerobic sequencing batch reactor (AnSBR) treating protein-rich wastewater

**DOI:** 10.1016/j.ese.2023.100296

**Published:** 2023-07-05

**Authors:** Zhe Deng, Julian Muñoz Sierra, Ana Lucia Morgado Ferreira, Daniel Cerqueda-Garcia, Henri Spanjers, Jules B. van Lier

**Affiliations:** a Delft University of Technology, Faculty of Civil Engineering and Geosciences, Stevinweg 1, 2628 CN, Delft, the Netherlands; b Veolia Water Technologies Techno Center Netherlands B.V. - Biothane, Tanthofdreef 21, 2623 EW, Delft, the Netherlands; c KWR Water Research Institute, Groningenhaven 7, P.O. Box 1072, 3430 BB, Nieuwegein, the Netherlands; d Institute of Ecology. A.C, Cluster Cientifico y Tecnologico BioMimic®, Carretera Antigua a Coatepec 351, El Haya, 91073, Xalapa, Veracruz, Mexico

**Keywords:** Anaerobic digestion, Anaerobic sequencing batch reactor (AnSBR), Protein, Microbial community, Slaughterhouse wastewater

## Abstract

Treating protein-rich wastewater using cost-effective and simple-structured single-stage reactors presents several challenges. In this study, we applied an anaerobic sequencing batch reactor (AnSBR) to treat protein-rich wastewater from a slaughterhouse. We focused on identifying the key factors influencing the removal of chemical oxygen demand (COD) and the settling performance of the sludge. The AnSBR achieved a maximum total COD removal of 90%, a protein degradation efficiency exceeding 80%, and a COD to methane conversion efficiency of over 70% at organic loading rates of up to 6.2 g COD L^−1^ d^−1^. We found that the variations in both the organic loading rate within the reactor and the hydraulic retention time in the buffer tank had a significant effect on COD removal. The hydraulic retention time in the buffer tank and the reactor, which determined the ammonification efficiencies and the residual carbohydrate concentrations in the reactor liquid, affected the sludge settleability. Furthermore, the genus *Clostridium sensu stricto 1*, known as protein- and lipids-degraders, was predominant in the reactor. Statistical analysis showed a significant correlation between the core microbiome and ammonification efficiency, highlighting the importance of protein degradation as the governing process in the treatment. Our results will provide valuable insights to optimise the design and operation of AnSBR for efficient treatment of protein-rich wastewater.

## Highlights

• The AnSBR achieved 90% of total COD removal for protein-rich wastewater treatment.
• Organic loading rate and hydraulic retention time were key operation parameters.
• The degradation of proteins and carbohydrates affected the sludge settleability.
• Protein degradation governed the process and correlated with *Clostridium* abundance.

## Graphical abstract

**Figure:**
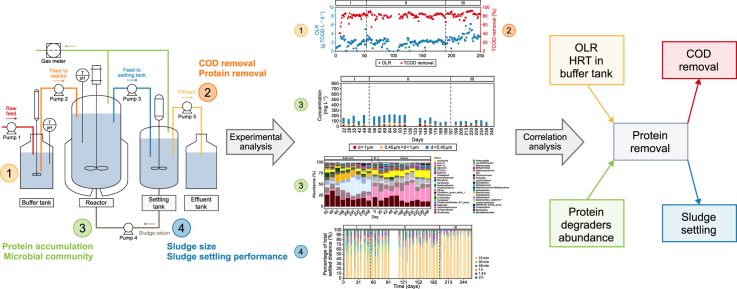

## 1 Introduction

The wastewater from several industries, such as the production of specific beverages and fish, dairy, and meat processing, contains large quantities of proteins that can account for up to 90% of the chemical oxygen demand (COD) [1]. Continuous increases in meat and meat alternatives production to meet the growing populations' protein needs have resulted in pollution issues [2,3]. For example, slaughterhouse wastewater (SWW) is characterised by high concentrations of organic compounds, such as proteins as well as fats, oil and grease (FOG), and high nutrient loads [4,5]. Direct discharge of SWW to surface water severely affects the environment and public health.

Current treatment methods include physiochemical and biological technologies [6,7]. Although anaerobic digestion (AD) is generally preferred for treating wastewater with a high organic load, a treatment chain, including an anaerobic high-rate reactor, typically starts with pre-treatment to decrease the high concentrations of solids and fats [4]. The major challenge in the treatment of SWW is the presence of high contents of proteins and FOG, which commonly accumulate in single-stage reactors [4,6]. Additionally, various undesirable effects, such as foaming, sludge flotation, sludge washout, and inhibition caused by the accumulation of long-chain fatty acids (LCFAs) from lipid degradation, can occur during the treatment [8,9]. These obstacles make it difficult to treat high-strength SWW efficiently. Development of a simple single-stage method for effectively treating SWW at acceptable costs is challenging [10,11]. In a previous study, we found that carbohydrates were degraded before proteins and that the constant presence of carbohydrates retarded the degradation of proteins [12].

Under anaerobic conditions, proteins are first hydrolysed to peptides and amino acids, then deaminated to ammonium (NH_4_^+^) and volatile fatty acids (VFAs). This process is also known as ammonification. Accumulation of ammonium to concentrations exceeding 1500 mg L^−1^ can cause inhibition, especially to methanogens, at the commonly applied reactor pH and temperature [13]. Breure et al. [14] suggested that the degradation of proteins and carbohydrates should be separated in space or time. Such separation can be accomplished using the feast-and-famine operation of an anaerobic sequencing batch reactor (AnSBR), allowing for the depletion of more rapidly degradable compounds and using intervals for the degradation of more slowly degradable compounds. Tan et al. [15] compared the effects of different feeding regimes on the performance of anaerobic membrane bio-reactors and found that the accumulation of proteins and carbohydrates was 30–50% higher in a continuously-fed reactor than in a batch-fed reactor.

AnSBRs combine feeding, bioconversion, settling, and decanting processes within a single reactor, and are beneficial for SWW treatment because of their low capital costs and low energy and manpower requirements [16]. The AnSBR is considered an alternative to continuous-flow single-stage reactors, such as anaerobic filters and upflow anaerobic sludge blanket reactors, which experience clogging, biomass adhesion issues, granule breakage and loss of density, scum formation, and sludge flotation and washout [17]. Various combinations of pre- and post-treatment, different cycle sequences, organic loading rates (OLR), and hydraulic retention times (HRT) have been designed and tested [10]. According to Shende and Pophali [18], an OLR range between 1.1 and 12.8 g COD L^−1^ d^−1^ can be applied in conventional AnSBRs with a total cycle sequence duration of 24–42 h and a reaction time longer than 16 h. An AnSBR can achieve a total COD removal efficiency between 78% and 97%. However, to achieve high COD removal efficiencies, the HRT, which is usually coupled with the reaction time, needs to be long compared with other high-rate anaerobic technologies, such as upflow anaerobic sludge blanket reactors and anaerobic baffled reactors. Additionally, because of batch-wise feeding, a parallel reactor is required for continuous flow operation [18]. Moreover, poor sludge settling performance leads to deterioration in the effluent quality, while settling of the resulting flocculent sludge often takes a considerable time, thereby reducing the reaction time [19].

The anaerobic bioconversion process largely depends on the activity and balanced metabolic cooperation of the microbiome in the reactor. The presence of specific microbiota is determined by operational parameters, such as the pH, OLR, and HRT, and may ultimately affect the performance of the reactor [20]. According to Ziels et al. [21], the feeding frequency and OLR are the two main parameters that exert a selective pressure and influence the microbial community structure and biokinetic conversion of complex substrates in AnSBRs.

Given the theoretical advantage of AnSBRs for protein-rich wastewater treatment, the aim of our study was to evaluate the treatment performance of an AnSBR to treat SWW regarding COD removal, protein degradation, overall conversion of COD to methane (CH_4_), and sludge settling. The applied AnSBR consisted of a main reactor followed by a settling tank. Consequently, the reaction time in the reactor was not limited by the required settling time for the cultivated sludge. In fact, with the applied set-up, the reaction time lasted for the entire batch cycle period. The SWW was fed directly to the AnSBR without additional physiochemical pre-treatment. Three operation phases with various OLRs and HRTs were applied to assess the process performance under different conditions. Correlation analysis between the operational parameters and performance indicators was performed to identify the processes determining the operational parameters. Moreover, we investigated the microbial community dynamics and diversity while paying particular attention to the microbiome of protein-degrading amplicon sequence variants (ASVs) and their correlation with performance and operational parameters. This study will provide valuable insights to optimise the design and operation of AnSBR, ensuring efficient treatment of protein-rich wastewater.

## 2 Methods and materials

### 2.1 Inoculum and wastewater characteristics

The inoculum sludge was taken from an anaerobic high-rate granular sludge bed reactor of a biochemical company producing pharmaceuticals in the Netherlands. The sludge had a total suspended solids (TSS) content of 9.2 g L^−1^ and a volatile suspended solids (VSS) content of 8.4 g L^−1^. An initial sludge concentration of 10 g VSS L^−1^ in the reactor was obtained by filtering 36 L of inoculum.

The protein-rich feed to the AnSBR was raw wastewater collected from the inlet of an SWW treatment plant (HydroBusiness B.V., Breda, the Netherlands). The main characteristics of the wastewater and inoculum are shown in Table 1.

**Table 1:** Characteristics of protein-rich SWW and inoculum.

Parameters	Units	SWW	Inoculum
pH	-	6.8–7.8	-
TSS	mg L^−1^	1100–3700	9200
VSS	mg L^−1^	1100–3500	8400
TCOD	mg L^−1^	4700–6500	-
SCOD	mg L^−1^	1500–3800	-
NH_4_^+^-N	mg L^−1^	175–420	-
Proteins^a^	mg L^−1^	500–1150	-
Carbohydrates^b^	mg L^−1^	100–250	-
FOG	mg L^−1^	100–600	-

a The protein concentration was measured as mg BSA L^−1^,which has a conversion factor of 1.5 g COD per g BSA.

b The carbohydrate concentration was measured as mg glucose L^−1^,which has a conversion factor of 1.1 g COD per g glucose.

### 2.2 Reactor setup and operational conditions

**Fig. 1:**
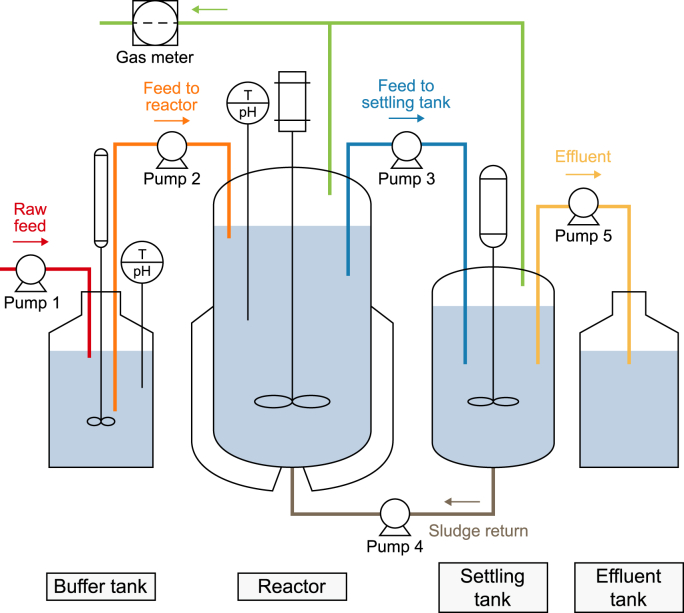
Schematic representation of the AnSBR experimental setup.

The AnSBR set-up consisted of a 10-L buffer tank (BT), a 30-L reactor, and a 12-L settling tank (Fig. 1). The treated effluent was collected in an effluent tank. Four cycles were performed each day, and each cycle lasted for 6 h. At the start of each cycle, the effluent of the BT was fed to the reactor, after which the BT was replenished with raw SWW. Two hours after feeding, 10 L of the reactor content was transferred to the settling tank for degassing (2 h) and settling (2 h). At the end of the 2-h settling phase, the liquid in the settling tank was discharged to the effluent tank, and the settled sludge was returned to the reactor.

The OLR was calculated as the daily COD load for the four cycles by multiplying the influent total COD (TCOD) concentration by the added volume and dividing it by the reactor working volume. The reactor OLR was gradually increased from 2.0 g COD L^−1^ d^−1^ to the designed threshold of 6.2 g COD L^−1^ d^−1^ over three different operational phases. During the start-up phase (phase I, days 0–55), the biomass was acclimated to the SWW. In phase II (days 56–196), the reactor was operated with an average OLR of 2.0 ± 0.6 g COD L^−1^ d^−1^ and an average solid loading rate (SLR) of 0.2 ± 0.1 g COD per g VSS per d. In phase III (days 197–260), the OLR was gradually increased to 6.2 g COD L^−1^ d^−1^ to investigate the maximum treatment capacity of the reactor. The pH in the BT and the reactor was kept between 6.8 and 7.5 using a controller (SC200 Universal Controller, Hach, Loveland CO, USA) equipped with pH sensors (K8, Hamilton, Denmark). The temperature of the reactor was controlled at 36 ± 0.5 °C with a recirculating water bath (MX06S135, VWR International, Radnor PA, USA). The BT and the settling reactor were operated at ambient temperature (20 ± 5 °C). The operational parameters of the reactor, including the average HRT and solid retention time (SRT), are summarised in Table 2. The HRT was calculated by dividing the reactor volume by the average daily flow rate, which was the total added volume divided by 24 h. The SRT was calculated by dividing the total sludge (g) in the reactor by the daily wasted sludge (g d^−1^).

**Table 2:** Loading rate, retention time, solids content and cycle time in the buffer tank, reactor, and settling tank.

AnSBR	OLR^a^	SLR	HRT	SRT	TSS	VSS	Cycle Time^b^
	(g TCOD L^−1^ d^−1^)	(g TCOD per g VSS per d)	(d)	(d)	(g L^−1^)	(g L^−1^)	(h)
Buff tank	2–6	-	0.7 ± 0.5	-			6
Reactor^c^	I: 1.8 ± 1.0	0.25 ± 0.13	2.9 ± 1.6	55 ± 15	7.0 ± 1.0	6.0 ± 1.0	4
	II: 2.0 ± 0.6	0.17 ± 0.07	2.4 ± 1.3	115 ± 35	8.8 ± 1.1	7.9 ± 1.1	4
	III: 3.2 ± 1.0	0.23 ± 0.06	1.5 ± 0.7	60 ± 10	10.6 ± 2.0	9.9 ± 2.0	4
Settling							
tank	-	-	-	-	-	-	2

a The OLR was calculated as the daily organic load of the four cycles, i.e., influent TCOD concentration times added volume, divided by the reactor working volume.

b Four cycles a day.

c Averaged values of each stage with standard deviation are shown, the temperature was maintained at 20 ± 5 °C in the buffer tank and 36 ± 0.5 °C in the reactor, pH was regulated in the range of 6.8–7.5.

### 2.3 Sampling and analysis

#### 2.3.1 Basic analytical parameters

Samples were taken from the raw feed, BT, reactor, and effluent tank. The TCOD and soluble COD (SCOD) were analysed twice a week. The TCOD and SCOD measurements were carried out using HACH-Lange kits (LCK014, Merck Life Science NV., the Netherlands). Samples were filtered through a 0.45-μm membrane filter (WHA10463513, Whatman, Delft, the Netherlands) before SCOD measurement. TSS, VSS, total Kjeldahl nitrogen (TKN), soluble Kjeldahl nitrogen, NH_4_^+^, and VFAs were analysed once a week. The solids content and nitrogen concentrations were analysed following standard methods [22]. Samples for VFA analysis were first centrifuged at 13,500×*g* for 5 min and then filtered through 0.45-μm membrane filters (WHA10463513, Whatman). The VFAs composition was analysed as described by Tan et al. [15].

The COD removal efficiencies (%) were calculated as the difference in COD concentration between the influent and effluent, divided by the COD concentration in the influent. The acidification degree (%) in the BT was calculated as the percentage of VFA production, expressed in g COD, from the influent TCOD or SCOD. The ammonification efficiency (%) was calculated as the NH_4_^+^ concentration (mg N L^−1^) divided by the TKN concentration (mg N L^−1^) in the BT and reactor multiplied by 100.

Protein and carbohydrate concentrations were analysed once a week. Samples from the BT and the reactor were centrifuged at 6500×*g* for 10 min and subsequently filtered with 1- or 0.45-μm membrane filters (WHA10463523 and WHA10463513, Whatman), after which the protein and carbohydrate contents were measured. The protein concentrations were assessed following the manufacturer's protocol with the bicinchoninic acid kit (BCA1-1 KT, Merck Life Science NV.). The absorption was measured by a spectrometer at 562 nm. Bovine serum albumin was used as a standard. Carbohydrates were analysed following the Dubois method [23].

Biogas production was monitored by a drum-type gas meter (TG05/5, Ritter, Germany), and the biogas composition, including the percentages of CH_4_, CO_2_, and O_2_, was analysed using a BIOGAS 5000 Analyzer (Scantec Industries NV, Belgium). The COD to CH_4_ (${COD}_{{CH}_{4}}$) conversion efficiency (%) was calculated as the CH_4_ production (in g COD d^−1^) relative to the COD fed to the reactor (in g COD d^−1^) using the following equation:

(1) $${COD}_{{CH}_{4}}\;conversion\;efficiency\;\left(\%\right)=\frac{{CH}_{4-COD}\;production\;rate}{COD\;fed\;to\;reactor}$$

where the CH_4-COD_ production rate is the daily CH_4_ production (g COD d^−1^), and the COD fed to the reactor is the daily organic matter fed to the reactor (g COD d^−1^).

The sludge's particle size distribution (PSD) in the 0.01–2000 μm range was measured using a Microtrac Bluewave light scattering instrument (Retsch Technology GmbH, Germany) with Microtrac FLEX 11.1.0.2 software. The flow rate was 25%.

#### 2.3.2 Settling distance and zone settling velocity

The settling distance of the sludge was assessed by measuring the distance from the surface of the liquid to the liquid–solid interface at 15 min, 30 min, 45 min, 1.5 h, and 2 h. The zone settling velocity (ZSV) was used to characterise the sludge settleability. The unhindered settling velocity (*v*_0_) and the compressibility factor (*k*) were measured with a transparent vertical cylinder according to an established method White [24]. The ZSV was calculated using the Vesilind equation [25] as follows:

(2) $$v=v_{0}e^{-kX}$$

where *v* is the ZSV (m h^−1^), *X* is the sludge concentration (g TSS L^−1^) in the reactor, *k* is the compressibility factor (L per g TSS), and *v*_0_ is the unhindered settling velocity (m h^−1^).

#### 2.3.3 AnSBR cycle analysis

Cycle analysis was performed by taking samples from the BT and the reactor every hour throughout one entire cycle (6 h). The samples were used for size fractionation analysis of COD, proteins, and carbohydrates, and the results were used to track the degradation of organic matter during the operation.

#### 2.3.4 Microbial community analysis

Sludge samples (10 mL) were taken regularly from the BT and the reactor and centrifuged at 13,500×*g* for 5 min. The supernatant was discarded and the biomass pellets were stored at −20 °C in Eppendorf tubes (Eppendorf, Germany). The sludge samples were then sent for DNA extraction and amplicon sequencing (Novogene, UK).

Before the DNA extraction, the biomass pellets were thawed, and biomass from duplicate samples was combined, weighed to 250 mg, and then transferred to PowerBead Pro tubes. DNA was extracted with the DNeasy PowerSoil Pro Kit (Qiagen, Germany), and the DNA quality and quantity were verified by agarose gel electrophoresis and using a 5400 Fragment Analyzer System (Agilent, USA). DNA (16S rRNA gene) amplification was carried out using the Illumina Novaseq 6000 platform. The primers were 341F [(5′–3′) CCTAYGGGRBGCASCAG] and 806R [(5′–3′) GGACTACNNGGGTATCTAAT] for bacteria/archaea in the V3–V4 regions.

The Illumina paired-end raw reads were processed using the QIIME2 platform [26]. The raw reads were inspected for their Phred quality scores to prune and trim low-quality positions. With the DADA2 plugin [27], raw reads were trimmed to position 20 at the 3′ end and truncated to position 240 in both the forward and reverse reads. The ASVs were resolved, and chimeric sequences were removed with the consensus method. The representative sequences of ASVs were taxonomically assigned with the “classify-consensus-vsearch” plugin [28], using the SILVA 138 database [29] as a reference. A phylogeny was built with the “align-to-tree-mafft-fasttree” plugin. Briefly, this plugin aligned the sequences with the MAFFT algorithm [30], then ambiguous positions were masked, and a tree was constructed with the FastTree2 algorithm [31]. The resulting abundance table and phylogeny were exported to the R environment.

Samples were normalised by rarefaction to 50000 counts. Alpha and beta diversities were calculated with the phyloseq library [32] in R. Taxonomic abundance was scaled to relative abundance (%) and visualised with the ggplot2 library [33]. A principal coordinate analysis was plotted to visualise the beta diversity differences between samples using the unweighted UniFrac distance metric.

To determine the shared ASVs in samples, 1000 random resamples were performed with replacing. In each resample, the ASVs that prevailed in all samples were detected. Only the ASVs that prevailed in 95% of all resamples were considered as the core microbiota. Core members within the operational phases were analysed with BLAST against the Refseq RNA database to identify the closest related species. The sequences were deposited in the SRA (NCBI) database under the accession number PRJNA847614.

#### 2.3.5 Statistical correlation analysis

Correlation analysis of different operational parameters was performed to identify the most relevant parameters affecting the performance of the reactor. The output variables were the TCOD and SCOD removal efficiencies and the ZSV. Pearson correlation coefficients were calculated to assess the correlations between two parameters, with *p* < 0.05 indicating a significant correlation. The correlations were categorised into four levels according to the *p*-values and the absolute values of the correlation coefficients. A *p*-value higher than 0.05 indicated that there was not a significant correlation between the two parameters. A *p*-value lower than 0.05 and an absolute coefficient value of 0.00–0.30 indicated that there was a low correlation between the two parameters, an absolute coefficient of 0.31–0.60 indicated a moderate correlation and an absolute coefficient of 0.61–1.00 indicated a high correlation.

A permutational multivariate analysis of variance (PERMANOVA) was calculated with the vegan library [34] to correlate the changes in the microbial community composition (distances) in samples from the AnSBR system with the operational parameters and performance indicators. The datasets were considered statistically different when a *p*-value of ≤0.05 was obtained.

## 3 Results and discussion

### 3.1 AnSBR performance

#### 3.1.1 Treatment efficiencies under varied operational conditions

##### 3.1.1.1 COD removal efficiencies and conversion efficiencies

**Fig. 2:**
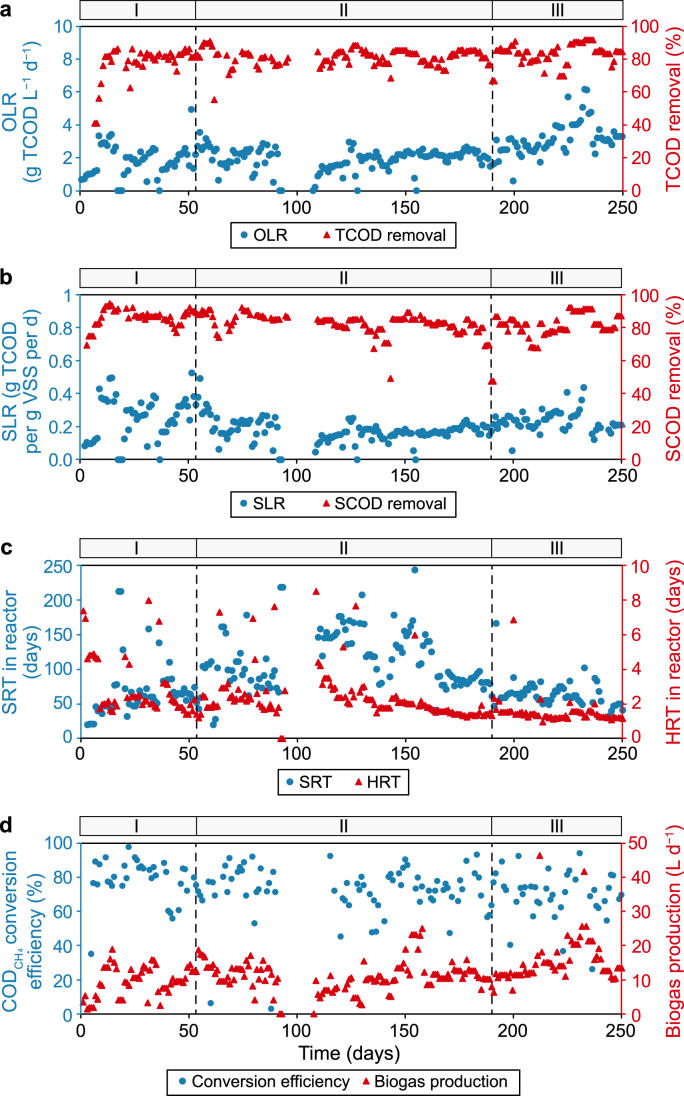
Operation and performance of the AnSBR set-up. **a**, OLR and TCOD removal efficiency. **b**, SLR and SCOD removal efficiency. **c**, SRT and HRT in the reactor during the operation period of 260 days. **d**, ${COD}_{{CH}_{4}}$ (COD to CH_4_) conversion efficiency of organic compounds to CH_4_ and daily biogas production.

The AnSBR was operated for 260 days. During the start-up phase (phase I) with an OLR of 1.8 ± 1.0 g TCOD L^−1^ d^−1^, the TCOD removal efficiency gradually increased and reached an average of 78 ± 10% (Fig. 2a). In the stable operation phase (phase II), an OLR of 2.0 ± 0.6 g TCOD L^−1^ d^−1^ was applied, and the average TCOD removal efficiency was 81 ± 5%. In the last operational phase (phase III) with an OLR of up to 6.2 g TCOD L^−1^ d^−1^, the TCOD removal efficiency reached an average of 83 ± 6%. A maximum removal efficiency of 90% was achieved at the highest OLR. The SCOD removal efficiency (Fig. 2b) was 87 ± 5% during phase I, then decreased to 82 ± 8% during phase II and 83 ± 6% during phase III as the OLR increased.

The SRT and HRT during the operational periods are shown in Fig. 2c. During phases I and II, the SRTs were 55 ± 15 and 115 ± 35 days, respectively. During phase III, the SRT gradually decreased to 60 ± 10 days. During phases I and II, the average HRTs were 2.9 ± 1.7 and 2.4 ± 1.3 days, respectively. In phase III, the HRT was 1.5 ± 0.7 days.

Hai et al. [35] reported a maximum removal efficiency of 98% with an OLR of 2.0–6.8 g COD L^−1^ d^−1^ and an HRT of 24 h in an anaerobic/aerobic intermittent sequencing batch biofilm reactor treating SWW. Similarly, Rajab et al. [3] reported a maximum removal efficiency of 97% at an OLR of 0.5–4.5 g COD L^−1^ d^−1^ and an HRT of 72 h in a two-stage anaerobic/aerobic sequencing batch reactor. For an AnSBR without pre-treatment, the recommended OLRs for treating diluted and concentrated SWW are 4.5 and 6.0 g COD L^−1^ d^−1^, respectively [36]. The applied operational cycle in our study, including feeding, reaction, settling, and decanting, took 6 h. Compared with single- and two-stage anaerobic/aerobic sequencing batch reactors with similar OLRs reported by Hai et al. [35] and Rajab et al. [3], our AnSBR set-up is considered compact and energy-efficient for treating SWW.

The daily biogas production (L d^−1^) and COD to CH_4_ (${COD}_{{CH}_{4}}$) conversion efficiency (%) are shown in Fig. 2d. The ${COD}_{{CH}_{4}}$ conversion efficiency was defined as the conversion of organic compounds to CH_4_ relative to the amount fed into the AnSBR. During phase I, the average ${COD}_{{CH}_{4}}$ conversion efficiency was 79 ± 12%. As the OLR increased, the conversion efficiency decreased to 72 ± 15% during phase II, and slightly decreased again to 70 ± 14% in phase III. Although the average OLRs were similar during phases I and II, there was less variation in the OLR, particularly in the final part of phase II (Fig. 2a). With a more stable OLR, there were fewer feed interruptions during the operation, which resulted in a higher total volumetric loading in phase II than in phase I. Consequently, the HRT, particularly in the later part of phase II, was shorter than in phase I (Fig. 2c). Apparently, the methanogenic biomass was negatively affected by the shortened HRT, which led to a decrease in the ${COD}_{{CH}_{4}}$ conversion efficiency.

##### 3.1.1.2 Degree of acidification of TCOD and SCOD in the BT

**Fig. 3:**
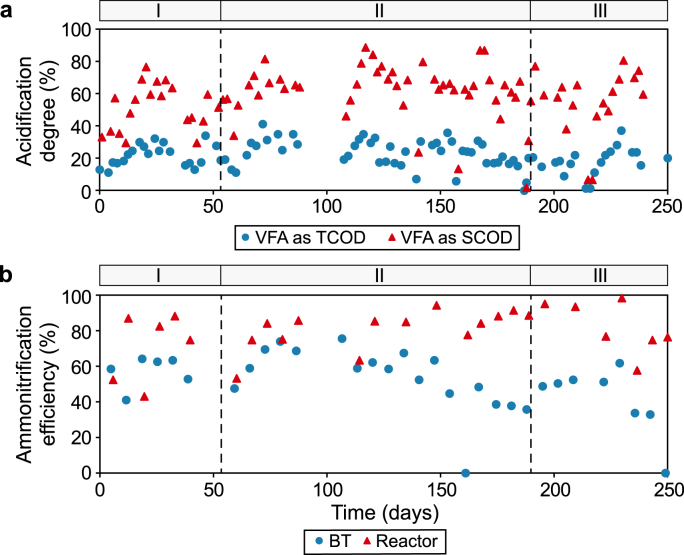
Acidification degree and ammonification efficiency. **a**, VFA production as percentages of TCOD and SCOD (i.e., acidification degree) in the buffer tank (BT). **b**, Ammonification efficiency (NH_4_^+^/TKN) in the BT and reactor.

Irrespective of the type of influent and the applied pre-treatment technology, an increase in SCOD is reported to improve the biogas production by a factor of 1.2–1.5 and the COD removal efficiency by a factor of 1.2–1.8 [37]. In the present study, a BT was applied to pre-acidify the raw SWW with the aim of enhancing hydrolysis and the availability of soluble substrates to the microorganisms in the reactor. The acidification degree (%) was used to indicate VFA production as a percentage of TCOD and SCOD in the BT. This was measured to evaluate the effect of pre-acidification on COD removal and the ${COD}_{{CH}_{4}}$ conversion efficiency. Fig. 3a shows the VFA production in the BT during the operational period. The acidification degree of TCOD was below 40%, which was likely because of the high solids content of the influent. The acidification degree of SCOD was generally higher than 60% after phase I. During phase III (days 196–260), the degree of pre-acidification was varied by adjusting the HRT in the BT, aiming for a high degree of pre-acidification in the BT.

##### 3.1.1.3 Ammonification efficiency in the BT and reactor

The ammonification efficiency was calculated as the measured ammonium concentration (mg N L^−1^) divided by the measured TKN concentration (mg N L^−1^) (Fig. 3b). During phase I, the average ammonification efficiency in the BT was 57 ± 9%. In the reactor, the ammonification efficiency increased from 50% to 85%, which indicated that the microbes were acclimated to the SWW, which was likely caused by the recirculation of the settled biomass. During phase II, the ammonification efficiency decreased to an average of 53 ± 18% with increases in the OLR in the BT. In the reactor, the ammonification efficiency increased to an average of 82 ± 12%. Between days 125 and 200, the ammonification efficiency increased to 92 ± 12%. During phase III, the ammonification efficiency in the BT decreased to an average of 47 ± 10%. In the reactor, the ammonification efficiency decreased from >90% to <80% as the OLR increased from 3.5 to 6.2 g COD L^−1^ d^−1^. The average ammonification efficiency during phase III was 87 ± 20%. Other research has shown that protein degradation is greatly retarded under a high OLR (e.g., 20–25% ammonification with >100 g COD L^−1^ d^−1^) [38]. According to our results, a moderate OLR between 2.0 and 3.5 g COD L^−1^ d^−1^ is recommended for maintaining a high ammonification efficiency (≥85%). The NH_4_^+^-N/TKN ratios were similar in the raw SWW and the BT, which indicated that proteins were mainly degraded in the reactor.

##### 3.1.1.4 Proteins and carbohydrates in the BT and reactor liquid

**Fig. 4:**
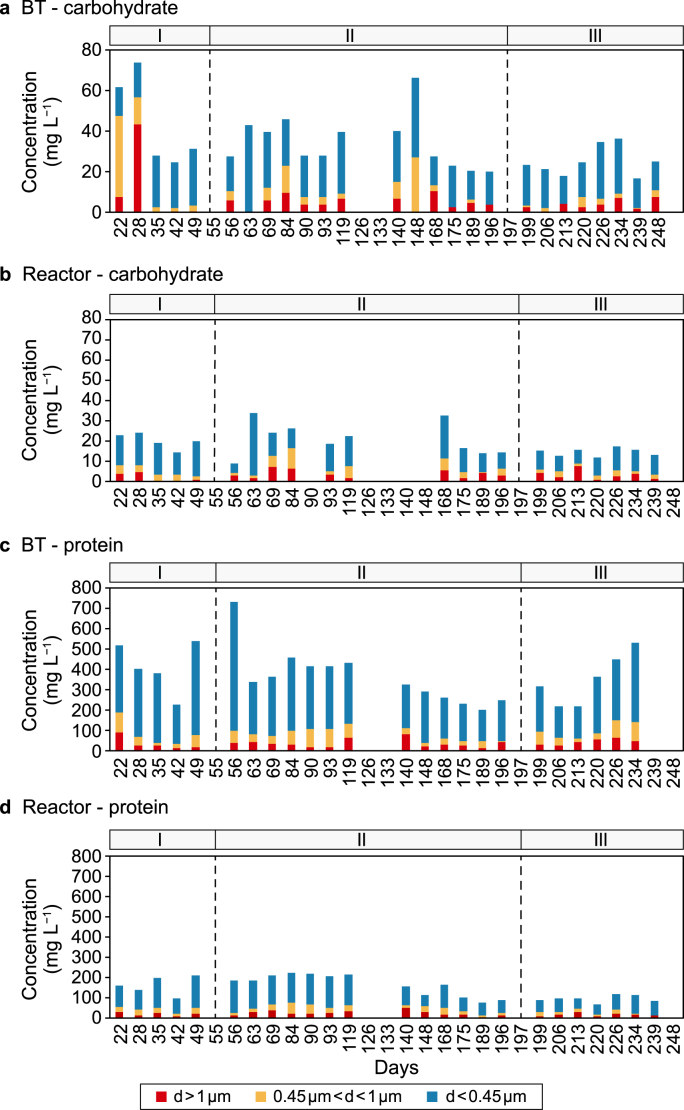
Concentration of carbohydrates and proteins. **a**–**b**, Three size fractions of carbohydrates concentrations in buffer tank (BT) (**a**) and reactor liquid (**b**). **c**–**d**, Three size fractions of proteins concentrations in buffer tank (**c**) and reactor (**d**). Averaged results of hourly samples in one cycle were shown, and the different size fractions were indicated by different diameters, the x-axis indicates the operational days.

Cycle analysis was performed once a week. Samples of the BT and reactor liquids were collected every hour during one cycle to investigate the system performance. Fig. 4 shows three size fractions of carbohydrates and proteins (>1 μm, 1–0.45 μm, and <0.45 μm), including suspended, colloidal, and soluble matter. The results were averaged and compared between the BT and reactor liquid.

Generally, carbohydrates and proteins that are smaller than 1 μm were dominant in the BT and reactor liquid, contributing to 40–90% of the total concentrations. The carbohydrate concentration decreased from an average of 40 mg L^−1^ in the BT liquid to an average of <20 mg L^−1^ in the reactor liquid (Fig. 4a,b). In phase III, the carbohydrate concentration in the reactor remained below 15 mg L^−1^ despite the higher OLR, which indicated that there was active removal of carbohydrates in the reactor.

The protein concentration in the BT varied between 200 and 750 mg L^−1^ (Fig. 4c). From day 22 to 109, the protein concentration decreased to below 200 mg L^−1^ in the reactor (Fig. 4d). It then further decreased to <100 mg L^−1^ in the second half of phase II. Decreases in the concentrations of the three-size fractions of proteins were observed throughout the operational period. The observed decreases were attributed to the ongoing acclimatisation of the microbes to the feed wastewater, which would also explain the increase in ammonification efficiency. The protein concentration remained at approximately 100 mg L^−1^ regardless of the OLR during phase III.

The applied AnSBR showed efficient degradation of proteins for treating real SWW. Anaerobic protein degradation is reportedly often in the range of 17–77% [39]. Additionally, the concentration of proteins accumulated in an AnSBR reactor liquid may increase from 250 to 1500 mg L^−1^ after an increase in the OLR from 3.1 to 5.5 g COD L^−1^ d^−1^ [15]. In the present study, the AnSBR achieved an average protein degradation efficiency of 81 ± 10% at an OLR of 2.0–6.2 g COD L^−1^ d^−1^, and the protein concentration was kept below 100 mg L^−1^ in the reactor. To further improve the protein degradation efficiency, granular sludge with a higher conversion capacity could be used, which would reduce the concentration of all organic residues [18].

#### 3.1.2 Sludge settling performance

**Fig. 5:**
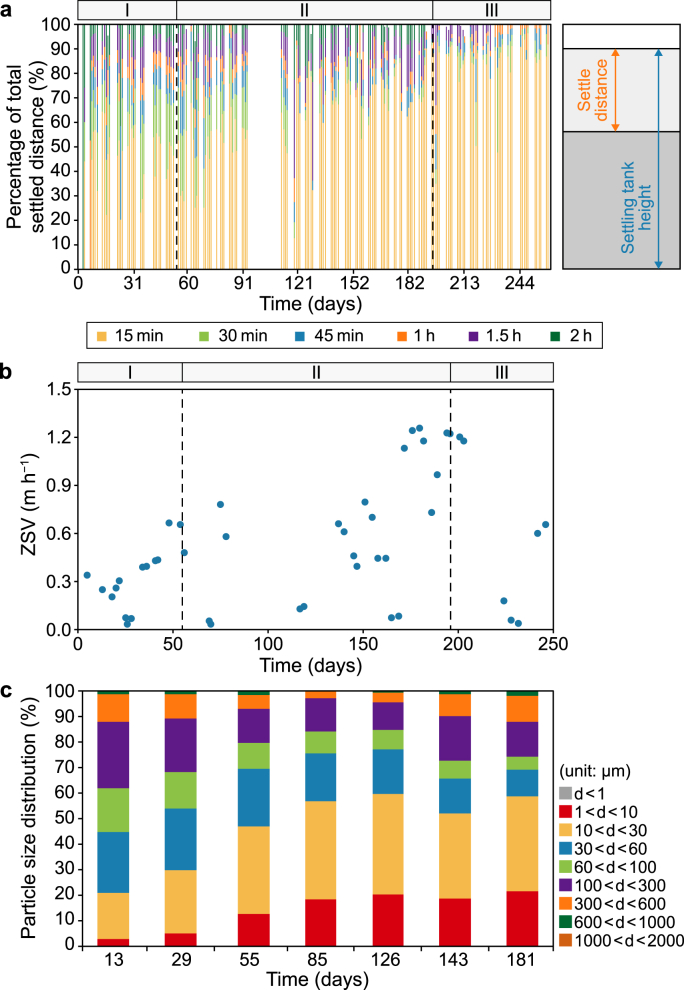
Sludge settling performance. **a**, Settled sludge distance in different time periods. **b**, Zone settling velocity (ZSV) of the sludge. **c**, Particle size distribution (PSD) of the sludge in reactor during the start-up phase and stable operation phase, *d* is mean diameter of the particle (μm), the averaged results of three samples are presented in one column.

The settling distances of the sludge in the first 15 min, 30 min, 45 min, 1.5 h, and 2 h as 100% stacked bars are plotted in Fig. 5a. During phase I, approximately 50% of the sludge settling distance was achieved within the first 15 min, and 75% was reached within the first 30 min. The settling was improved during the more stable AnSBR operation of phase II, with 80% of the sludge settling distance achieved within the first 15 min. A settling time of 45 min or longer only led to an increase of approximately 20% in the settling distance. The sludge settling was further improved during phase III, with more than 90% of the sludge settled within the first 15 min. However, the total settling distance decreased from 55% of the settling tank height during phase I to 45% during the stable operation phase (phase II), and further decreased to 35% during the high-loading phase (phase III).

The settling behaviour of sludge is considered to be an essential performance indicator for reactors that use gravitational separation of solids from liquid [40]. In the present study, the settling distance within the first 15 min clearly connected to the ZSV. The lowest ZSVs occurred on days 26, 70, 119, 169, and 232, and also when short settling distances within the first 15 min were observed (Fig. 5a,b). The settling distance analysis could be used to set a required settling time during an operational cycle, with the ZSV being indicative of the sludge settling performance. *Ex situ* assessment of the ZSV is particularly useful when the settling distance in full-scale reactors cannot be measured.

In addition to the sludge settleability, the particle size distribution (PSD) and the mean particle size may affect sludge sedimentation, thickening, digestion, and subsequent dewatering [41]. Therefore, we collected samples to monitor the PSD in the reactor and investigate the correlation between PSD and sludge settleability. The PSD results showed the following: (1) a steady increase in the fraction of small particles (1 μm < diameter <30 μm); (2) a steady decrease in the fraction of particles with diameters between 30 and 300 μm; and (3) an initial decrease in the fraction of large particles (300 μm < diameter <600 μm) with increases in the SRT, followed by an increase in this fraction with decreases in the SRT (Figs. 2c and 5c).

An average SRT exceeding 50 days in phase I and II decreased the fraction of 300–600 μm particles and increased the fraction of 1–30 μm particles. However, the increase in the fraction of 1–30 μm particles did not negatively affect the sludge settling or ZSV. In phase I, 55% of the sludge was settled within 15 min, and the fraction of small particles increased from 20% to 45% (Fig. 5a). The ZSV increased during phase I, and when the reactor was restarted on day 111, the ZSV increased from <0.3 to 1.2 m h^−1^, while the fraction of small particles remained at 50% (Fig. 5b). Hence, the variation in PSD was not correlated with the sludge settling performance during the operational period.

### 3.2 Microbial community dynamics and diversity

**Fig. 6:**
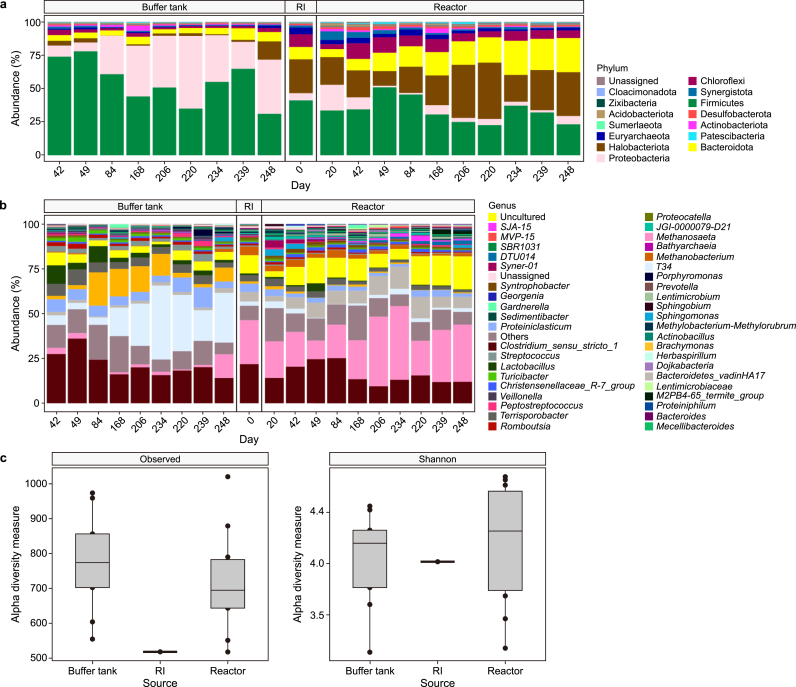
**a**–**b**, Microbial community dynamics in the buffer tank, inoculum (RI), and reactor at the phylum level (**a**) and genus level (**b**). Phase I: up to day 55, phase II: up to day 197, and phase III: up to day 260. **c**, Alpha diversity plots for the microbial community in the buffer tank, RI, and reactor. Left: observed ASV numbers; Right: Shannon's index.

The structure and dynamics of the microbial community in the BT and the reactor biomass were analysed (Fig. 6) and possible correlations with protein degradation in the reactor were assessed. The dominant bacteria in the BT belonged to the phylum Firmicutes, followed by Proteobacteria and Bacteroidota (Fig. 6a). The relative abundance of Firmicutes decreased from 76 ± 3% in phase I, to 52 ± 12% in phase II and 47 ± 11% in phases III. For Proteobacteria, the relative abundance increased from 8 ± 1% in phase I to 38 ± 11% in phase III. The relative abundance of Bacteroidota remained at approximately 5 ± 1% throughout the operational period, and all belonged to the class Bacteroidia (Fig. S1). Bacteroidia play a crucial role in the degradation of complex polymers and are proteolytic bacteria involved in converting proteins to VFAs and ammonium [42]. The most abundant genera were *Clostridium sensu stricto 1* (21 ± 7%) and *T34* (19 ± 13%), and, especially in phase III*, Brachymonas* (9 ± 7%)*, Proteiniclasticum* (6 ± 2%), *Terrisporobacter* (5 ± 2%), *Lactobacillus* (4 ± 3%), *Methanosaeta* (3 ± 4%), *Streptococcus* (3 ± 1%), *Bacteroidetes vadinHA17* (2 ± 1%), and *Romboutsia* (2 ± 1%) (Fig. 6b). *Clostridium sensu stricto 1* metabolises diverse compounds present in SWW, such as proteins/amino acids*,* carbohydrates, and short-chain fatty acids [43], and has a major role in lipid/LCFAs degradation and a syntrophic relationship with methanogens [44]*.* A high protein content combined with low fatty acid content favours the abundance of *Proteiniclasticum*, which can also degrade amino acids and proteins [45]. *Terrisporobacter* has high hydrolytic capabilities [46]. *Romboutsia* species are anaerobes adapted to nutrient-rich environments, in which carbohydrates and sources of amino acids and vitamins are abundant [47].

During the entire operation, the most dominant bacteria in the reactor belonged to the phyla Firmicutes (34 ± 9%), Bacteroidota (17 ± 7%), Chloroflexi (8 ± 3%), and Proteobacteria (6 ± 5%), while the dominant archaea were Halobacteriota (26 ± 10%) and Euryarchaeota (3 ± 1%) (Fig. 6a). Jabari et al. [48] also reported that the most frequently detected bacteria in an anaerobic reactor treating protein-rich SWW belonged to Firmicutes, specifically to the class Clostridia (Fig. S1), and Bacteroidota. As in the BT, *Clostridium sensu stricto 1* was also prevalent in the reactor but decreased in relative abundance from 18 ± 4% in phase I to 13 ± 2% in phase III as the OLR increased. By contrast, the abundance of the archaea Methanosaeta increased from 22 ± 3% to 30 ± 9% during phase III (Fig. 6b), which indicated that enrichment of methanogens occurred in the reactor. The species enriched with the highest similarity were *Methanothrix harundinacea strain 8Ac* and *Methanothrix soehngenii GP6* (Table S1).

It should be noted that the identified core microbiome contained mainly protein/amino acids degraders such as *Turicibacter* sp., *Turicibacter sanguinis strain MOL361*, *Romboutsia* sp., *Romboutsia timonensis strain DR1*, *Proteiniclasticum*, *Clostridium sensu stricto 1* sp., and *Clostridium disporicum strain DS1* in the BT, and *Proteiniclasticum* and *Clostridium sensu stricto 1* in the reactor (Fig. S2). Other protein- and amino acid-degrading genera such as *Proteocatella* and *Proteiniphilum* were also present but with lower relative abundances (Fig. 6b). The dominance of protein degraders indicated that the AnSBR was favoured for selection of this microbiota.

Alpha diversity indices were used to compare the evenness and richness of the microbial population of the inoculum and in the BT and reactor throughout the entire operational period (Fig. 6c). The median alpha diversity metrics from the observed ASVs were 775 for the BT, 696 for the reactor, and 525 for the inoculum. Even though the BT had the highest microbial richness, the variation among the scores indicated no substantial differences among the samples. By contrast, the Shannon index showed a higher score in the reactor (4.27) than in the BT (4.16) or inoculum (4.02) (Fig. 6c). Because the Shannon index score considers the richness and evenness of the microbial population, the highest value observed in the reactor indicated a more even microbial population than in the BT. The differences in the diversity among the samples were attributed to the changes in the OLR over 260 days. These changes could promote higher diversity but could also lead to variable microbial community functions [49]; that is, a high alpha diversity may indicate a relatively stable COD conversion in the reactor. Still, a decreasing COD to CH_4_ conversion efficiency was observed even though the enrichment of methanogens occurred. Apparently, there was no increase in methanogenic activity, which might also be attributed to reasons other than microbial diversity and species richness. Moreover, beta diversity analysis of the principal coordinates indicated that the reactor's microbial community matched the inoculum sample and differed from that in the BT (Fig. S3).

### 3.3 Correlation analysis

#### 3.3.1 Parameters affecting COD removal

Pearson correlation analysis showed that the SLR, HRT, SRT, and acidification degree of TCOD had low correlations (<0.30) with the TCOD removal efficiency (Table 3). Therefore, variations in these parameters would have a minor effect on TCOD removal. At the same time, the OLR, acidification degree of SCOD, and ammonification efficiency in the reactor had moderate correlations (0.31–0.60) with the TCOD removal efficiency. Among the identified indicators, the ammonification efficiency in the reactor had the highest correlation coefficient (0.56) with TCOD removal. The results showed that protein degradation had a statistically significant contribution to TCOD removal, and the ammonification efficiency was a good indicator of protein degradation.

**Table 3:** Correlation analysis of TCOD, SCOD, ZSV, reactor operational, and performance parameters.

Output variable	Pearson correlation	Loading rate		HRT	SRT	Acidification		Ammonification		Protein concentration	Carbohydrate concentration
		OLR	SLR			TCOD	SCOD	BT	Reactor	Reactor liquid	Reactor liquid
TCOD removal	Correlation coefficient^a^	0.37	0.25	−0.17	0.17	0.27	0.31	0.08	0.56	0.10	0.02
	*p*-value^b^	0.00	0.00	0.01	0.01	0.01	0.00	0.68	0.00	0.64	0.93
SCOD removal	Correlation coefficient	0.30	0.41	0.08	0.00	0.60	0.38	0.20	−0.20	0.51	0.03
	*p*-value	0.00	0.00	0.21	0.98	0.00	0.00	0.29	0.30	0.01	0.90
ZSV	Correlation coefficient	0.15	0.01	−0.40	−0.05	−0.36	−0.03	−0.71	0.7	−0.39	−0.81
	*p*-value	0.32	0.93	0.01	0.76	0.08	0.88	0.03	0.05	0.39	0.03

a For correlation coefficient: 0.00–0.30 low correlation; 0.31–0.60 moderate correlation; 0.61–1.0 high correlation.

b For *p*-value: no correlation when *p* > 0.05.

SLR, acidification, and protein concentration in the reactor liquid showed moderate correlations (0.31–0.60) with the SCOD removal efficiency. SLR had a correlation coefficient of 0.41, and OLR had a correlation coefficient of 0.30 with SCOD removal. Acidification of TCOD had a correlation coefficient of 0.60 with SCOD removal, which meant that hydrolysis and/or acidification of TCOD was limiting SCOD removal. The protein concentration in the reactor liquid had a correlation coefficient of 0.51 with SCOD removal, which indicated that residual protein in the reactor was limiting SCOD removal.

Our results indicate that the OLR in the reactor and HRT in the BT, which determines the acidification degree, should be prioritised as control parameters when treating SWW with a high solids content. However, when treating wastewater containing mainly SCOD, the SLR in the reactor and HRT in the BT should be prioritised as control parameters.

Overall, both the loading rate and attained acidification degree in the BT are important for COD removal. Additionally, the ammonification efficiency in the reactor is closely related to TCOD removal, and protein residues in the reactor liquid affect SCOD removal. The applied ranges of HRT and SRT in the reactor do not significantly affect TCOD and SCOD removal, indicating that the applied retention times are sufficiently long. Therefore, further investigations are required to determine the correlation between the COD removal efficiency and applied retention time and to optimise the required reactor volume.

#### 3.3.2 Parameters affecting the sludge ZSV

There were high correlations between the sludge ZSV and ammonification efficiency in the BT (−0.71) and reactor (0.70), and between the ZSV and the carbohydrate concentration in the reactor liquid (−0.81) (Table 3). These results indicated that the degradation of proteins and carbohydrates had a significant effect on the sludge settling performance. The high negative correlation (−0.71) between ammonification efficiency in the BT and the ZSV illustrates that a shorter HRT in the BT is preferable for better settling. The HRT in the reactor had a moderate correlation with the ZSV, and the negative value indicated a higher HRT in the reactor led to a lower ZSV. Possibly, an increase in the fraction of small particles in the sludge at a high HRT will gradually contribute to a reduction in the ZSV over long-term operation.

The correlation analysis identified the HRT in the BT and the reactor as the key parameter affecting sludge settling. A relatively short HRT in the BT and reactor is recommended to achieve a higher ZSV. However, the HRT in the BT also controls the acidification of TCOD and SCOD, which have low–moderate correlations with TCOD and SCOD removal. Further studies are needed to optimise the HRT in the BT and the reactor to achieve both high COD removal and good sludge settling.

#### 3.3.3 Microbial community correlation

A PERMANOVA statistical test (Table 4) was carried out to analyse the correlation between the core microbial community structure, the AnSBR performance indicators, and the operational parameters. A statistically significant correlation (*p* < 0.05) was identified between the core microbial community and the attained ammonification efficiency (*R*^2^ = 0.14). The *R*^2^ of PERMANOVA represents the correlation of the distance matrix with a given variable, which is the variance or the difference in composition between samples explained by this variable. The results indicated that the change in the core microbial community structure, specifically the variation in the relative abundance of the protein-degrading *Clostridium sensu stricto 1* (Fig. S2b), affected the ammonification efficiency. Even though the coefficient of determination was low, it inferred that protein ammonification was the key process governing the AnSBR treatment performance. Similarly, Li et al. [50] found that *Clostridium sensu stricto*, the dominant microbiome genus in 20 full-scale anaerobic reactors treating manure, was positively correlated with increasing ammonium concentrations in the reactors. Moreover, Duong et al. [1] concluded that degradation of amino acids to NH_4_^+^-N, VFAs, H_2_, and CO_2_, instead of hydrolysis of proteins to amino acids, governed optimisation of the design for anaerobic reactors treating protein-rich wastewater.

**Table 4:** Statistical significance between the microbial community and ammonification efficiency in reactor by PERMANOVA (*p* < 0.05) analysis (using unweighted unifrac distance).

Factor	*R*^2^^a^	*p*-value^b^
TCOD removal (%)	0.12	0.29
SCOD removal (%)	0.11	0.48
OLR (g COD L^−1^ d^−1^)	0.12	0.29
SLR (g COD per g VSS per d)	0.10	0.76
HRT (d)	0.09	0.80
SRT (d)	0.14	0.05
Acidification (VFAs) TCOD (%)	0.10	0.75
Acidification (VFAs) SCOD (%)	0.11	0.44
Ammonification efficiency (%)	0.14	0.01

a *R*^2^: coefficient of determination.

b *p*-value: probability value.

## 4 Conclusions

Without pre-removal of FOG or solids, the present AnSBR set-up achieved a maximum TCOD removal of 90%, a protein degradation efficiency exceeding 80%, and a COD to methane conversion efficiency of over 70% at OLRs up to 6.2 g COD L^−1^ d^−1^ when treating protein-rich SWW. The OLR substantially affected the COD removal efficiency of the AnSBR, concomitantly with the HRT in the BT and the SLR. The HRT in the BT and the reactor, which determined the ammonification efficiencies and the residual carbohydrate concentrations in the reactor liquid, affected the sludge settleability. Furthermore, the genus *Clostridium sensu stricto 1*, involved in protein and lipid degradation, was predominant in the reactor. Additionally, the core microbiome showed a low but statistically significant correlation with the ammonification efficiency in the reactor, which indicated that the degradation of proteins and amino acids was the governing process determining the overall COD removal and reactor performance.

## CRediT authorship contribution statement

**Zhe Deng**: Conceptualization, Investigation, Data Curation, Formal Analysis, Visualization, Writing - Original Draft. **Julian Muñoz Sierra**: Conceptualization, Methodology, Formal Analysis, Visualization, Writing - Original Draft. **Ana Lucia Morgado Ferreira**: Conceptualization, Investigation, Data Curation, Writing - Review & Editing. **Daniel Cerqueda-Garcia**: Software, Formal Analysis, Visualization, Writing - Review & Editing. **Henri Spanjers**: Supervision, Writing - Review & Editing, Supervision. **Jules B. van Lier**: Supervision, Writing - Review & Editing, Supervision.

## Declaration of competing interests

The authors declare that they have no known competing financial interests or personal relationships that could have appeared to influence the work reported in this paper.
