# Supplementary material for: Effect of operational parameters on the performance of an anaerobic sequencing batch reactor (AnSBR) treating protein-rich wastewater
Source: Environ Sci Ecotechnol. 2023 Jul 5;17:100296. doi: 10.1016/j.ese.2023.100296 (PMC10405192; doi:10.1016/j.ese.2023.100296)
Supplement: Multimedia component 1 [file mmc1.docx]

**Effect of operational parameters on the performance of an anaerobic sequencing batch reactor (AnSBR) treating protein-rich wastewater**

Authors

*Zhe Deng^a, b^

Julian Muñoz Sierra^a,c^

Ana Lucia Morgado Ferreira^b^

Daniel Cerqueda-Garcia^d^

Henri Spanjers^a^

Jules B. van Lier^a^

*Corresponding author: z.deng-2@tudelft.nl

Author addresses

^a^ Delft University of Technology, Faculty of Civil Engineering and Geosciences, Stevinweg 1, 2628 CN Delft, the Netherlands

^b^ Veolia Water Technologies Techno Center Netherlands B.V. - Biothane, Tanthofdreef 21, 2623 EW Delft, The Netherlands

^c^ KWR Water Research Institute, Groningenhaven 7, P.O. Box 1072, 3430 BB Nieuwegein, the Netherlands

^d^ Institute of Ecology. A.C, Cluster Cientifico y Tecnologico BioMimic®, Carretera Antigua a Coatepec 351, El Haya, 91073 Xalapa, Veracruz, Mexico.

**Supplementary Material**

**
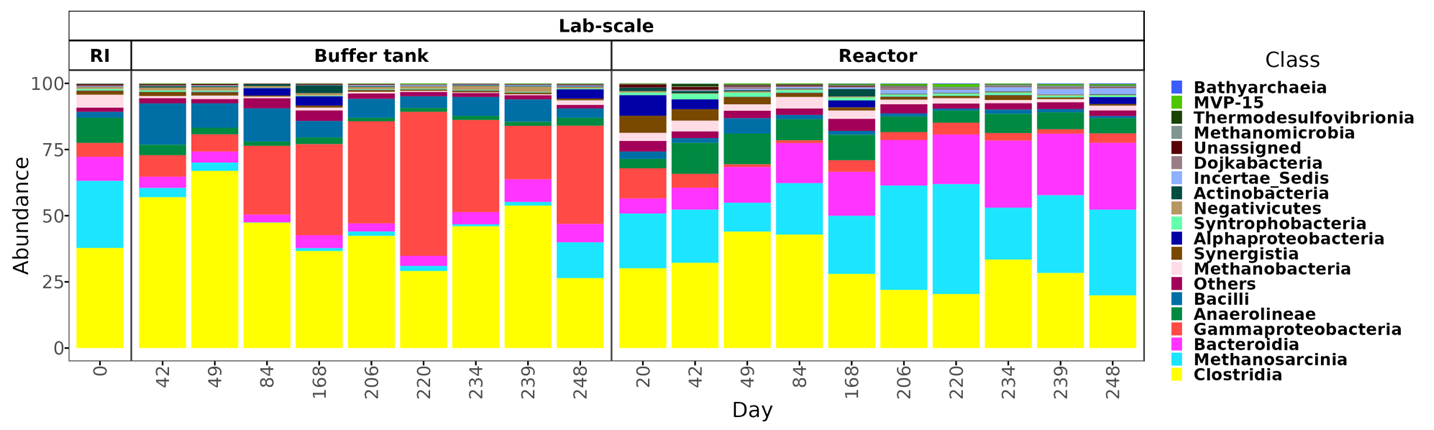
**

Fig. S1 Microbial community dynamics in AnSBR and buffer tank at the class level.


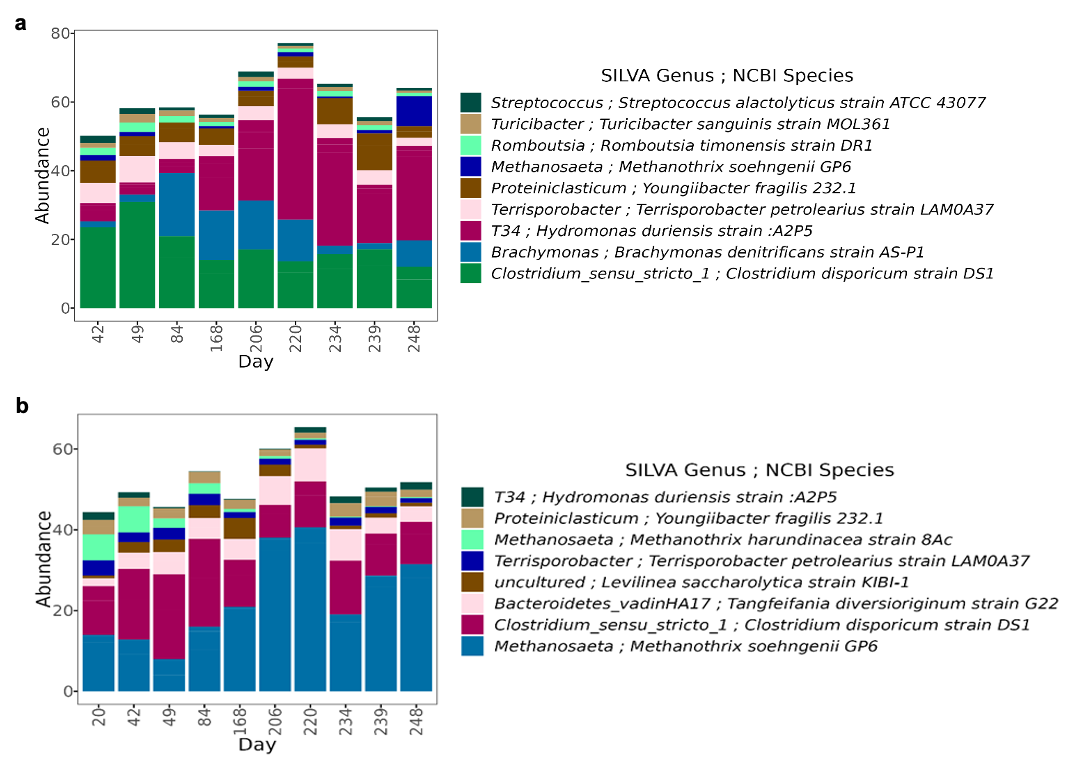


Fig. S2 Core microbiome or predominant amplicon sequence variants (ASVs) with average relative abundance > 1% in A) Buffer tank and B) Reactor at the species level.

| 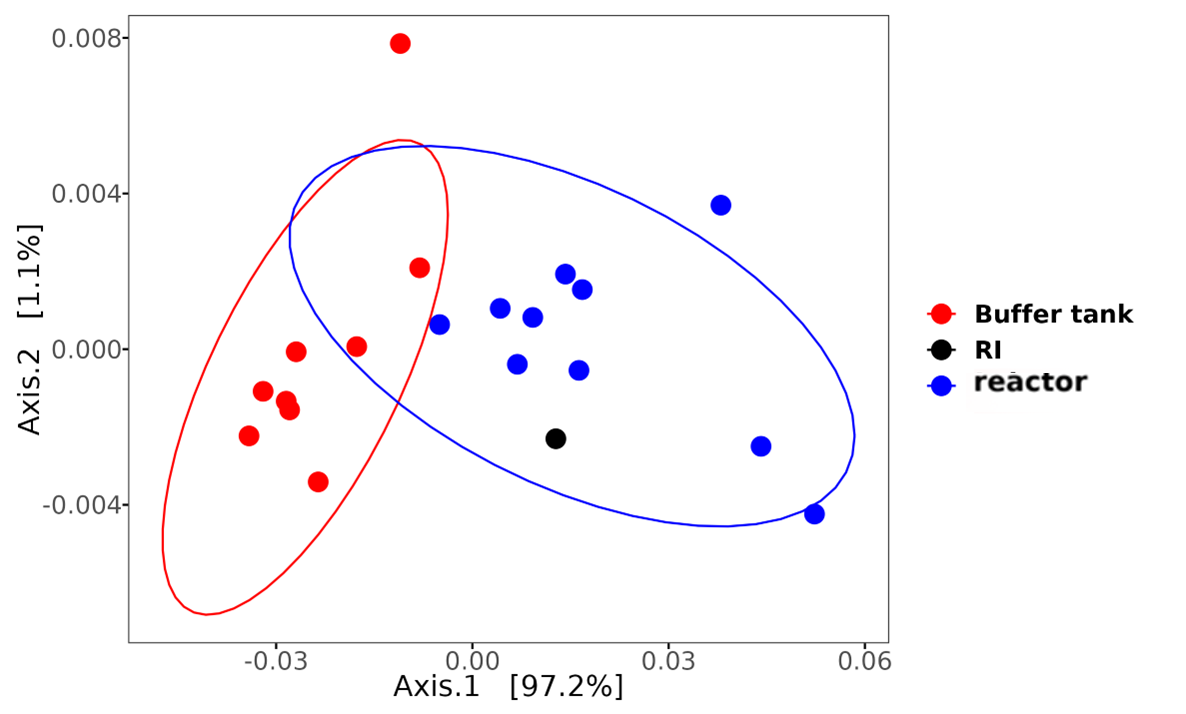 |
| --- |

Fig. S3 Beta diversity PCoA analysis among buffer tank and reactor samples.

**Table S1** Most prevalent genera in AnSBR and buffer tank and their best BLAST similarity hits with species reported in the NCBI taxonomy.

| Accesion | Similarity E-value | SILVA Genus | NCBI taxonomy | Source |
| --- | --- | --- | --- | --- |
| NR_040972.1 | 88.9 | *uncultured* | *Levilinea saccharolytica strain KIBI-1* | AnSBR |
| NR_118108.1 | 96.4 | *Proteiniclasticum* | *Youngiibacter fragilis 232.1* |  |
| NR_026491.1 | **100.0** | *Clostridium_sensu_stricto_1* | *Clostridium disporicum strain DS1* |  |
| NR_137408.1 | **99.3** | *Terrisporobacter* | *Terrisporobacter petrolearius strain LAM0A37* |  |
| NR_043203.1 | **99.7** | *Methanosaeta* | *Methanothrix harundinacea strain 8Ac* |  |
| NR_102903.1 | **100.0** | *Methanosaeta* | *Methanothrix soehngenii GP6* |  |
| NR_102903.1 | 96.9 | *Methanosaeta* | *Methanothrix soehngenii GP6* |  |
| NR_102903.1 | 97.5 | *Methanosaeta* | *Methanothrix soehngenii GP6* |  |
| NR_145650.1 | 96.1 | *T34* | *Hydromonas duriensis strain :A2P5* |  |
| NR_118108.1 | 94.9 | *Proteiniclasticum* | *Youngiibacter fragilis 232.1* |  |
| NR_134211.1 | 88.2 | *Bacteroidetes_vadinHA17* | *Tangfeifania diversioriginum strain G22* |  |
| NR_134211.1 | 86.9 | *Bacteroidetes_vadinHA17* | *Tangfeifania diversioriginum strain G22* |  |
| NR_118108.1 | 96.4 | *Proteiniclasticum* | *Youngiibacter fragilis 232.1* | Buffer tank |
| NR_026491.1 | 98.3 | *Clostridium_sensu_stricto_1* | *Clostridium disporicum strain DS1* |  |
| NR_026491.1 | **100.0** | *Clostridium_sensu_stricto_1* | *Clostridium disporicum strain DS1* |  |
| NR_041781.1 | **100.0** | *Streptococcus* | *Streptococcus alactolyticus strain ATCC 43077* |  |
| NR_028816.1 | **99.1** | *Turicibacter* | *Turicibacter sanguinis strain MOL361* |  |
| NR_137408.1 | **99.3** | *Terrisporobacter* | *Terrisporobacter petrolearius strain LAM0A37* |  |
| NR_144740.1 | **100.0** | *Romboutsia* | *Romboutsia timonensis strain DR1* |  |
| NR_102903.1 | 96.9 | *Methanosaeta* | *Methanothrix soehngenii GP6* |  |
| NR_145650.1 | 96.1 | *T34* | *Hydromonas duriensis strain :A2P5* |  |
| NR_145650.1 | 94.9 | *T34* | *Hydromonas duriensis strain :A2P5* |  |
| NR_145650.1 | 95.7 | *T34* | *Hydromonas duriensis strain :A2P5* |  |
| NR_118108.1 | 94.9 | *Proteiniclasticum* | *Youngiibacter fragilis 232.1* |  |
| NR_025834.1 | **100.0** | *Brachymonas* | *Brachymonas denitrificans strain AS-P1* |  |
